# Epigenetic crosstalk in neuroblastoma development and progression

**DOI:** 10.3389/fcell.2026.1769372

**Published:** 2026-03-11

**Authors:** Nathan Drolet, Amber Wolf, Joanna Yi, Eveline Barbieri

**Affiliations:** Department of Pediatrics, Section of Hematology-Oncology, Texas Children’s Cancer and Hematology Center, Baylor College of Medicine, Houston, TX, United States

**Keywords:** epigenetics, neural crest, neuroblastoma, oncogenesis, tumor microenvironment

## Abstract

Neuroblastoma (NB), a pediatric malignancy of the peripheral nervous system, accounts for 15% of pediatric cancer deaths and remains a major challenge for cancer therapy. NB originates from sympathoadrenal (SA) progenitors of the neural crest (NC) cell lineage, whose formation and differentiation are controlled by epigenetic mechanisms. Although signaling pathways and epigenetic factors implicated in NB tumorigenesis have been well-described, a holistic understanding of how development and epigenetics overlap to drive NB is lacking. By exploring vital interplays and co-dependencies between key pathways and their epigenetic control in NC development, we have gained novel insights into the mechanisms governing NB differentiation and oncogenesis. These converging insights have enabled the application of targeted therapies against epigenetic regulators driving NB with promising implications for patient outcomes.

## 1 Introduction

**FIGURE 1:**
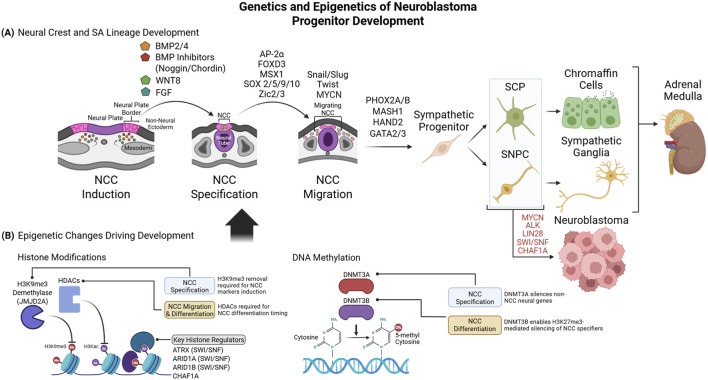
Genetics and Epigenetics of Neuroblastoma Progenitor Development **(A)**. Signaling ligands and their antagonists form gradients to induce activation of NCC-specific transcriptional programs at the neural plate border. NCCs are then driven into migration, while mitogenic factors maintain their progenitor state. Lastly, lineage-specific proteins promote NCC differentiation into a sympathoadrenal fate. NCC-derived Schwann cell precursors (SCPs) and sympathetic neural progenitor cells (SNPCs) differentiate into chromaffin and neuronal components of the adrenal medulla. Aberrant retention of mitogenic factors or failed differentiation signals can maintain a permanent stem-like state, causing NB **(B)**. Epigenetic changes enable stage-specific TF expression and subsequent phenotypic changes during NCC lineage development. Epigenetic writers and erasers drive transcriptional changes via histone readers that displace nucleosomes or recruit other nucleosome remodelers to permit or prohibit transcription. DNA methylation refines NCC development, by suppressing genes associated with alternative cell lineages or by inactivating stage-specific NCC genes once their stage is passed. Created in BioRender https://BioRender.com/me02w0m.

NB is caused by dysregulated differentiation of sympathetic nerve progenitor cells during normal development (Matthay et al., 2016), beginning in early neural formation stages. Segregation of the primary germ layers during gastrulation creates a dorsal ectoderm zone that specifies into neuroectoderm, forming the neural plate (Milet and Monsoro-Burq, 2012), which subsequently invaginates and closes to form the neural tube (Figure 1A). Neuroectoderm along the plate borders and dorsal neural tube specify into neural crest cells (NCCs) (Bronner and LeDouarin, 2012), driven by signals such as bone morphogenetic protein (BMP), wingless/INT1 (WNT), and fibroblast growth-factor (FGF) ligands secreted from surrounding ectoderm and mesoderm (Rogers et al., 2012). NCCs then migrate from the dorsal side of the tube to distant sites, yielding a diverse range of tissues, including peripheral nerves and glia, facial cartilage, and melanocytes (Dupin et al., 2018). NCC subpopulations along the prospective trunk migrate ventrally, generating dorsal root ganglia from the spinal cord and suprarenal sympathetic nerves, as well as Schwann cells and endocrine chromaffin cells within the adrenal medulla (Furlan et al., 2017). Malignant transformation of such sympathoadrenal (SA) lineage progenitors is believed to give rise to NB. Mediating these developmental events are heritable epigenetic perturbations, most notably methylation of DNA and nuclear histone tails, as well as non-heritable alterations to histone acetylation and RNA methylation, which collectively dictate transcription through chromatin restructuring. The plasticity of these epigenetic processes facilitates NB tumor initiation and progression via silencing of tumor-suppressors and activation of cell survival and mitogenic genes. In this Review, we discuss cell signaling, transcriptional, and epigenetic events that drive NC development and NB transformation. We also discuss how such epigenetic mechanisms crosstalk with critical environmental factors, including cell metabolism and immunity.

## 2 Regulation of NCC induction, specification, and migration

Every step of NCC development is controlled by distinct programs and transcriptional circuits (Figure 1A). NC induction from neuroectoderm is initially cued by gradients of BMPs and BMP inhibitors from adjacent mesoderm and non-neural ectoderm, driving NC determination along the neural plate (Nguyen et al., 1998) and controlling NCC migration (Sela-Donenfeld and Kalcheim, 1999). WNT and FGFs serve as additional inductive signals (Milet and Monsoro-Burq, 2012) to generate NCCs and derivatives from the neural plate (LaBonne and Bronner-Fraser, 1998). Additional signals then refine the pattern of NC development and lineage specificity, such as Notch and Delta-like-ligand (DLL) (Wakamatsu et al., 2000) and Sonic Hedgehog (SHH) (Selleck et al., 1998).

Transcription factors (TFs) integrate upstream cell-signaling events to drive NC specification, migration, and differentiation. Among the essential TFs for NC development is AP-2α (TFAP2A), which is induced by specific BMP gradient levels along the neural plate (Luo et al., 2003) and is necessary for normal NC tissue development and craniofacial structure formation (Zhang et al., 1996). Mechanistically, TFAP2A mediates H3K27ac (mark of active enhancers) deposition at NC enhancers (Rada-Iglesias et al., 2012) to drive nucleosome remodeling and expose binding sites for additional TFs, such as OCT4 and SOX2 (Hovland et al., 2022). Another BMP-induced NC specifier, the transcriptional repressor MSH homeobox 1 (MSX1), enforces NC initiation (Monsoro-Burq et al., 2005) by driving expression of the NC markers FOXD3, Slug, and Snail (Liem et al., 1995; Tribulo et al., 2003). SRY-box TF (SOX) family members are also key in driving early NC development: SOX9 is expressed in NCCs from neurulation through progenitor differentiation and is required for neural tube folding, expression of NC markers, and NC-derived cartilage development (Spokony et al., 2002). SOX10 is localized to trunk NCCs, persists longer in migrating NCCs (McKeown et al., 2005), and is induced subsequent to SOX9 in most neural tube regions (Cheung and Briscoe, 2003), driving NCC formation at the dorsal neural tube edge (Honore et al., 2003) and mediating differentiation of trunk NCC derivatives (Reiprich et al., 2008). SOX5 and SOX2 play equally important roles in inducing NC genes in cooperation with other NCC TFs (Hovland et al., 2022; Nordin and LaBonne, 2014), exemplifying the conserved but non-redundant functions of SOX family members in NC development. Lastly, certain Zic-family TFs–such as Zic1 – comprise another class of known NC regulators (Merzdorf, 2007), which are activated by specific BMP levels (Garnett et al., 2012) and enable the formation of NC-derived nerve tissues (Plouhinec et al., 2014). These NCC specification regulators are succeeded by another set of TFs (Slug, Snail, and Twist) that control NCC epithelial-to-mesenchymal transition (EMT) allowing detachment from the neural tube and migration to their target destinations (Aybar et al., 2003). Taken together, these overlapping waves of NC specifiers and EMT regulators link NC induction signals to transcriptional, differentiation, and migration phenotypes that underlie NC development.

## 3 Differentiation of NCC into sympathoadrenal lineage

NCCs at the trunk region of the neural plate adopt diverse fates, including parasympathetic and digestive system neurons, and chromaffin cells within the adrenal gland medulla (Gammill and Roffers-Agarwal, 2010). Most chromaffin cells derive from Schwann cell precursors (SCPs) and trunk NCC progenitors that exit the neural tube late in NCC migration. SA development is controlled by TFs such as SOX8 and SOX10 (Kastriti et al., 2022) that induce expression of SA-lineage markers (such as tyrosine hydroxylase (TH) and dopamine beta-hydroxylase (DBH)) and the SA master regulator paired-like homeobox 2B (PHOX2B) (Reiprich et al., 2008). PHOX2B, PHOX2A, and MASH1 serve as predominant early regulators of SA lineage specification and are required for sympathetic nerve and chromaffin cell formation (Stanke et al., 2004). They are activated by SOX10 in migrating trunk NCC and form positive feedback loops to enforce each other’s expression, while promoting SA morphologic and transcriptional changes (Kim et al., 2003). PHOX2B also drives transcription of *HAND2* and *GATA2/3* (Tsarovina et al., 2004; Howard et al., 2000), TFs expressed within the SA neuroblast lineage (Jansky et al., 2021) and required for development of sympathetic ganglia and other NC cell types (Tsarovina et al., 2004). Concurrently with SA specification, MYCN is expressed in pre-ganglion neuroblasts to drive proliferation and self-renewal (Huang and Weiss, 2013; Kramer et al., 2016), prolonging the neuroblast expansion phase and driving premature NC migration and SA marker expression (Wakamatsu et al., 1997). Following SA progenitor differentiation, MYCN and PHOX2B are downregulated, allowing these cells to lose their proliferative progenitor properties in favor of quiescence. Aberrant failure to downregulate MYCN consequently blocks post-mitotic differentiation and maintains unrestrained proliferation of SA progenitors (Alam et al., 2009).

## 4 From sympathoadrenal NCCs to NB oncogenesis

NB was originally believed to derive from dysregulated SA neuroblasts due to their similarities in morphology and anatomical location (De Preter et al., 2006). However, recent NB single-cell analyses suggest that the transcriptional programs of non-MYCN-amplified (non-MNA) NB more closely resemble differentiated chromaffin cells (Dong et al., 2020), whereas the transcriptomes of MYCN-amplified (MNA) NB are more similar to an undifferentiated, transitory NCC-like state, with elevated cell-cycle and EMT genes expression and chromaffin markers such as *PHOX2B* (Dong et al., 2020). More recently, it has been shown that certain SCPs adopt a sympathetic neuroblast-like identity prior to chromaffin differentiation (Kameneva et al., 2021), suggesting that transient neuroblasts may be the likely source of NB, whose dysregulated developmental programming has endowed them with unchecked replicative potential. This is supported by single-cell time-course analyses of NB xenografts that revealed evolution in their transcriptional state between undifferentiated precursor-like to neuroblast-like, in both developmental directions (Villalard et al., 2024). Similarly, when clinical NB transcriptomes were mapped to developmental trajectories derived from a comprehensive human and murine trunk NC lineage set, most individual NB cells closely matched early or late stages of neuroblast identity, with a mix of mesenchymal- and SA-like transcriptional features (Xu et al., 2025). NB may therefore originate from SCP-derivative neuroblasts but can shift in identity following oncogenesis.

To diverge from regimented SA differentiation, NB must acquire broad deviations in gene programs that control malignant cell properties (Supplementary Table S1). NB profiling across stages of disease progression suggests that dysregulation of specific pathways is required for early neuroblast-like cells to evolve into more aggressive NB states, including upregulation of pathways promoting cell-cycle progression and replication stress response (Bonine et al., 2024). Chemotherapy drives further transcriptome remodeling, activating E2F/mTOR or NF-κB pathways to overcome cytotoxicity-induced death induction (Avitabile et al., 2022; Grossmann et al., 2024). Advanced NB stages were also found to display distinct global patterns of epigenetic DNA methylation, which correlated with transcriptional dysregulation of specific NB drivers such as *TERT* (Lalchungnunga et al., 2022), although it remains unclear whether these broad methylation patterns are required for NB progression. Other epigenomic features–such as histone methylation and acetylation–are likewise reorganized by NB oncogenesis and define subtype-specific identity, and will be discussed later in greater detail.

As NB descends from dysregulated NCCs, several proteins that control normal NCC induction, proliferation, and specification have been implicated in coordinating the aberrant transcriptional and epigenetic changes driving NB oncogenesis. Among the most potent NB drivers is MYCN. MYC-family TFs regulate cell-cycle (Ruiz-Perez et al., 2017), metabolic (Yoshida, 2020), and apoptotic (McMahon, 2014) programs across diverse cell types, drive proliferation of NCCs (Murphy et al., 2005), and maintain multipotency of specific adult stem tissues (Laurenti et al., 2008). While MYC serves a mitogenic role in many stem-cell types, MYCN is specifically required for neural progenitor cell (NPC) proliferation and prevents premature NPC differentiation (Knoepfler et al., 2002; Stanton et al., 1992; Chen and Guan, 2022). MYCN is active in early NCCs (Wakamatsu et al., 1997; Vize et al., 1990), balancing NCC proliferation and differentiation through regulation of cyclin-dependent kinase inhibitors (Zhang et al., 2016) and NC lineage specifiers such as *PHOX2B* and *MASH1* (Olsen et al., 2017). Our lab and others have demonstrated that MYCN is downregulated following migration and differentiation of NC progenitors (Tao et al., 2021; Vize et al., 1990; Wakamatsu et al., 1997). Consequently, it is believed that failed MYCN inactivation in NC progenitors blocks their differentiation and initiates NB. TH-driven ectopic MYCN drives NB formation in mice (TH-MYCN GEMM) (Weiss et al., 1997) by transforming neuroblastic cells (Hansford et al., 2004). MYCN over-expression in murine NCCs also drives NB formation (Olsen et al., 2017), albeit in conjunction with p53 mutation and an immune-compromised background. Lastly, NCCs with transgenic *MYCN* and anaplastic lymphoma kinase (*ALK*) *in utero* enable NB development (Cohen et al., 2020), altogether supporting that MYCN expression is sufficient to drive NB initiation.

**FIGURE 2:**
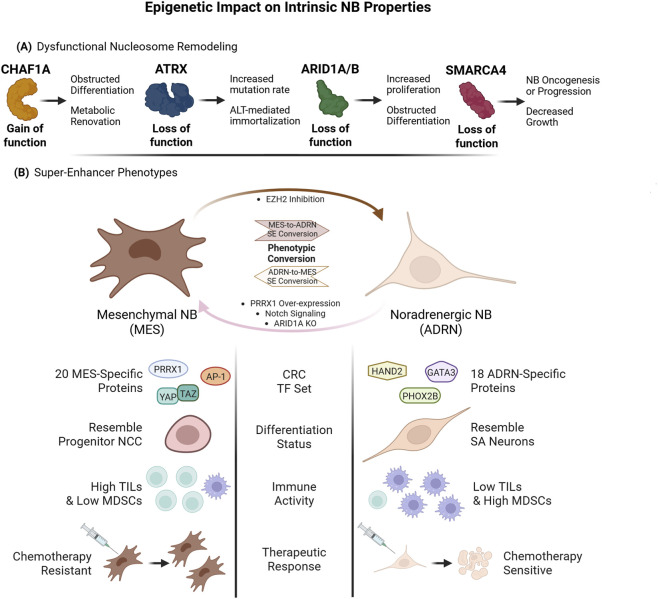
Epigenetic Impact on Intrinsic NB Properties **(A)**. Nucleosome remodelers are commonly mutated across NB subtypes. Gain-of-function activity by CHAF1A drives resistance to conventional RA-based differentiation therapies, mediated by metabolic overhaul that favors polyamine synthesis. Loss-of-function mutations in ATRX disrupt DNA damage repair, driving further mutation accumulation or ALT that confers immortalization. ARID1A/ARID1B mutations disrupt their control of tumor suppressors and differentiation-enforcing TFs in the NCC lineage. Finally, SMARCA4 loss-of-function is common in clinical NB but paradoxically is detrimental to NB cell growth **(B)**. H3K27ac at MES- and ADRN-specific CRC SEs controls CRC activation and thereby dictates NB phenotype. Each phenotype confers distinct properties, including different active TFs, a more undifferentiated-like and drug-resistant state in MES cells, and decreased immunogenicity in ADRN NB, each of which provides survival advantage in different contexts. Created in BioRender https://BioRender.com/g4ut8d5.

Chromatin remodelers also contribute to NB oncogenesis, by shifting, sliding, depositing and removing nucleosomes and histones (Figure 2A). These proteins use histone reader domains–such as H3K4me3-binding PHD fingers and H3K27ac-binding bromodomains–to recognize modified histone residues, and then hydrolyze ATP to twist DNA strands apart and reposition nucleosomes using their helicase domains (Patel and Wang, 2013). Several members of the SWItch/Sucrose Non-Fermentable nucleosome remodeler complex (SWI/SNF) regulate both NCC development and NB oncogenesis. The SWI/SNF subunit ARID1A is specifically required for NCC differentiation (Chandler and Magnuson, 2016), and its replacement in the BRG-/BRM-associated factor (BAF) variant of SWI/SNF by paralog ARID1B is necessary for iPSC differentiation into cranial NCCs (Pagliaroli et al., 2021). ARID1A knockout in NCCs maintains cell proliferation and blocks terminal differentiation (Garcia-Lopez et al., 2020). In NB, the 1p36.11 locus encompassing *ARID1A* is frequently deleted (Garcia-Lopez et al., 2020), and ARID1A or ARID1B loss-of-function mutations are detected in 10% of tumors, predicting worse clinical outcomes (Sausen et al., 2013). Combined haploinsufficiency of ARID1A and ARID1B enhances MYCN-driven NB tumorigenesis and TH-positive SA progenitor proliferation in DBH-MYCN zebrafish embryos (Shi et al., 2020; Li et al., 2017). In MNA tumors, ARID1A deletion increases cell survival and drives an adrenergic (ADR)-to-mesenchymal (MES) switch by reducing the binding of BAF to enhancers of genes that promote an ADR-like state, while increasing activity on MES genes (Jimenez et al., 2022; Shi et al., 2020). On the other hand, the NCC differentiation-enforcing agent retinoic acid (RA) upregulates ARID1A, driving recruitment of SIN3 TF member A (SIN3A) and suppressing telomerase reverse transcriptase (TERT) to permit differentiation (Bui et al., 2019).

Another chromatin remodeler–the nucleosome assembly factor Chromatin Assembly Factor 1A (CHAF1A) – controls expression of pluripotency master regulators *Oct4*, *Sox2* and *Nanog* in embryonic stem cells (ESC), and its expression is required for ESC differentiation into appropriate germ layers (Cheng et al., 2019). Our laboratory demonstrated that CHAF1A is co-expressed with MYCN in pre-migratory trunk NCC in zebrafish embryos, and both proteins are downregulated during NCC differentiation (Tao et al., 2021). CHAF1A over-expression targeted to NCCs inhibits zebrafish NCC differentiation and blocks RA-induced differentiation in an hESC-derived NCC model (Tao et al., 2021) (Figure 2A). Mechanistically, we found that ectopic CHAF1A activity drives NB growth and resistance to differentiation through its transcriptional induction of enzymes that synthesize polyamines–a metabolite class with known roles in DNA stability, gene regulation, and cell proliferation–notably, ornithine decarboxylase 1 (ODC1), which increased cellular levels of spermidine and spermine. Inhibition of polyamine synthesis via the ODC1 inhibitor DFMO reverses CHAF1A’s promotion of NB growth and RA differentiation insensitivity (Tao et al., 2021). Independently of CHAF1A modulation, polyamine disruption by DMFO has been shown to suppress NB proliferation by driving cell-cycle arrest (Gandra et al., 2024), and may extend clinical survival for both nascent and relapsed NB (Sholler et al., 2018), further suggesting that the induction of polyamine synthesis is a critical mechanism by which CHAF1A obstructs NB differentiation. Interestingly, spermidine drives *CHAF1A* transcription (Landau et al., 2012). This implies the existence of a positive-feedback loop connecting polyamine abundance to the expression of CHAF1A and other factors essential for DNA and cell replication, although further evidence is required to determine whether *CHAF1A* upregulation is required for spermidine’s ability to activate cell proliferation, and whether this holds true in NB. Since polyamine catabolism has been shown to reduce levels of acetyl-CoA and S-adenosylmethionine (Kee et al., 2004) – metabolites responsible for both transcription-activating and repressive chromatin modifications (Janke et al., 2015)– this also suggests that CHAF1A may indirectly regulate the epigenetic landscape via its induction of polyamine synthesis.

How does a nucleosome assembler produce such pleiotropic effects on transcription and metabolism? CHAF1A is known to regulate gene expression and differentiation in part through its secondary epigenetic function, recruiting epigenetic reader and writer proteins such as SETDB1 (Cheloufi et al., 2015), HP1α (Murzina et al., 1999), and histone deacetylases (HDACs) HDAC1/2 (Ng et al., 2019) to target loci. CHAF1A knockdown depletes transcriptionally repressive H3K9me3 and relieves chromatin compaction at developmental enhancers in de-differentiated fibroblasts (Cheloufi et al., 2015), and inactivation of its *Drosophila* homolog similarly decompacts PRC2-enforced heterochromatin (Song et al., 2007). CHAF1A also directly recruits TFs to mitogenic genes in cancer cells (Zheng et al., 2018), thus activating pro-growth and anti-differentiation pathways. Similar to MYCN, ectopic CHAF1A has been shown to suppress RA-induced transcription of neural differentiation markers in NB cells (Tao et al., 2021), whereas its depletion is sufficient to drive terminal differentiation of these cells (Barbieri et al., 2014). However, it remains unclear whether CHAF1A’s secondary epigenetic functions are responsible for its ability to suppress cell differentiation and modulate transcription of pro-growth metabolic effectors such as ODC1.

## 5 Epigenetic control of NCC and NB development

### 5.1 Histone modifications

Extensive research has characterized the NCC and NB epigenetic landscapes, including both classical epigenetic features that can be passed from mother to daughter cell, like DNA and histone methylation, and more transient features such as RNA methylation and histone acetylation that must be constantly maintained and renewed (Figure 1B). Among the key histone features driving NB development, H3K27ac deacetylation by HDACs 1-3 and 8 is a known requirement for trunk NCC specification, as obstruction of these HDACs prematurely upregulates NCC migration genes and blocks expression of differentiated lineage-specific genes, preventing appropriate temporal regulation of NCC migration and differentiation (Murko et al., 2013). Similarly, H3K9me3 levels increase globally in neural epithelia as they differentiate into early NCCs (Hu et al., 2014) but must be removed from NCC specification genes to permit NCC formation (Strobl-Mazzulla et al., 2010). Deposition of H3K4me1 – a mark often present alongside H3K27ac at active enhancers and promoters–is also essential for NCC formation and migration, as the H3K4 methyltransferase KMT2D is required for appropriate transcription of NC specifiers (Schwenty-Lara et al., 2020). Thus, coordinated remodeling, deposition, and removal of transcription-regulating histone modifications are necessary for proper NCC specification and differentiation.

Histone modifications are equally essential for NB development by regulating core regulatory circuit (CRC) enhancers that define NB phenotypes. NB is characterized by two distinct cell subtypes that dictate patient outcomes: the differentiated, chemo-sensitive, neuron-like ADRN cells and the undifferentiated, chemoresistant, hNCC-like MES cells (van Groningen et al., 2017; Gartlgruber et al., 2021) (Figure 2B). ADRN and MES NBs employ distinct CRC TF networks, which are activated by H3K27ac enrichment at their associated superenhancers (SEs) (van Groningen et al., 2017; Boeva et al., 2017). These TFs also regulate each other’s transcription via binding other CRC member’s SE regions in a positive-feedback mechanism, maintaining NB subtype identity (van Groningen et al., 2017). MYCN serves as a core TF for both ADRN and MES CRCs. Consequently, degradation of MYCN downregulates CRC expression and blocks NB tumor growth (Xu et al., 2023). Importantly, RA-induced differentiation drives permanent epigenetic inactivation of certain CRC-associated SEs in MNA NB (Gomez et al., 2022). Despite having distinct CRCs, ADRN and MES cells exhibit limited plasticity: over-expression of the MES-specific TF–PRRX1 – reprograms ADRN cells into MES (van Groningen et al., 2017), while over-expression of a NOTCH peptide drives an ADRN-to-MES transition by redistributing H3K27ac from ADRN-CRC to MES-CRC SEs (van Groningen et al., 2019). Additional studies are needed to uncover whether ADRN-to-MES or reverse transitions observed in relapsed NB are a transcriptional response to treatment, or whether expansion of existing sporadic MES or ADRN cells in heterogenous NB accounts for their greater prevalence.

Active SEs of the ADRN/MES CRCs contain DNA-bound TFs and epigenetic marks that drive transcription–including MYCN, H3K27ac, H3K4me1, and H3K4me3 – but lack repressive H3K27me3 and nucleosome occupancy (Upton et al., 2020). H3K27ac at these SEs is deposited by the p300/CBP complex, two closely related histone acetyltransferases (HATs) that drive TF binding, transcription initiation, and promoter-proximal polymerase pausing release (Narita et al., 2021). p300/CBP-mediated H3K27ac recruits bromodomain-containing BET family nucleosome remodelers–notably BRD4 – which in turn recruit P-TEFb to drive transcription elongation, as well as enlisting more TFs such as NF-kB (White et al., 2019). In cancer cells, p300/CBP is recruited to enhancers of oncogenes to drive their transcription, including *MYC* (Vannam et al., 2021; Raisner et al., 2018). Although p300 and CBP function almost exclusively as a complex in most cancers, ADRN NB employs p300 in a CBP-independent manner to upregulate SE-associated oncogenes (Durbin et al., 2022) and acetylates MYCN proteins to enhance their stability (Cheng et al., 2023). Importantly, this p300 dependency renders NB sensitive to its inhibition, such that p300 degradation by PROTAC degrader JQAD1 downregulates MYCN, reduces H3K27ac at ADRN CRC SEs, and upregulates pro-apoptotic programs (Durbin et al., 2022). H3K27ac depletion also mediates RA-induced proliferative arrest and terminal neuronal differentiation of NB cells (Zeid et al., 2018; Zimmerman et al., 2021). However, because histone modifications are reversible, studying epigenetic changes that emerge to drive relapse after p300 ablation or RA treatment needs further exploration.

### 5.2 Chromatin remodelers

Chromatin remodeling complexes read the “signals” of histone modifications to enable or block transcription (Becker and Workman, 2013). ATRX is required for neuronal differentiation and survival (Berube et al., 2005) and is mutated in approximately 10%–40% of NB patients depending on age group (Cheung et al., 2012). Due to its role in DNA damage repair, ATRX depletion in NB cells causes DNA damage accumulation and repair failure, which enables immortalization through alternative lengthening of telomere (ALT) activation, while rendering ATRX mutants sensitive to DNA-damaging agents (George et al., 2020). Heterozygous germline missense mutations and loss-of-function deletions in the ATPase subunit of the BAF and Polybromo-associated BAF (PBAF) SMARCA4/BRG1 have also been identified in NB patients (Witkowski et al., 2023); however, the functional consequences of these genetic variations and their clinical implications remain poorly understood (Bellini et al., 2019). SMARCA4 inactivation blocks NB growth and downregulates key regulators of cell survival (Jubierre et al., 2016), as well as dismantles TF occupancy and reduces accessibility at ADRN CRC SEs (Cermakova et al., 2023). SMARCA4 expression in NB is also controlled by SOX11 (Decaesteker et al., 2023), suggesting that the subversion of normal SA-lineage transcriptional programs in NB may support further nucleosome remodeling to drive cell survival. Thus, therapeutic strategies aiming at targeting SMARCA4 may restrict NB oncogenesis (Figure 2A). Another chromatin remodeler, BRD4, is required for robust MYCN expression in MNA NB (Puissant et al., 2013). BRD4 binds both the *MYCN* promoter and enhancer to drive its transcription, such that BRD4 inhibition precludes MYCN transcription, yielding anti-tumor activity in multiple MNA NB models (Henssen et al., 2016; Puissant et al., 2013). However, resistance to BET inhibitors can develop through epigenetic plasticity. Whereas normal cells maintain tight control over their epigenetic landscapes, cancer cells exhibit more stochastic changes in epigenetic marks and chromatin structure across their genomes, leading to spontaneous *de novo* gene activation and repression (Flavahan et al., 2017). In NB cells, BET inhibitor resistance is driven by epigenetic remodeling of SEs, including *de novo* activation of enhancers that upregulate the PI3K signaling rendering NB vulnerable to PI3K inhibition (Iniguez et al., 2018).

### 5.3 DNA methylation

Crosstalk between histone modifications and DNA methylation regulates cell differentiation during development. In ESCs, overlapping H3K27me3 and H3K4me3 at key genes is required to maintain a “poised” state, which are removed or reinforced to establish lineage-specific transcriptional programs in response to differentiation cues (Bernstein et al., 2006). DNA methylation and H3K27me3 is likewise employed to silence pluripotency-regulating genes and enforce lineage restriction as differentiation progresses (Mohn et al., 2008). Clinically, certain cancers utilize H3K4me3 and H3K27me3 bivalency at tumor-suppressor genes to silence their expression (Lin et al., 2015). The transcriptional malleability conferred by epigenetic bivalency also permits rapid tumor adaptation to therapeutics (Bapat et al., 2010). The DNA methyltransferase DNMT3A blocks non-NC specification of neuroepithelia by silencing *Sox2*/*3* expression (Hu et al., 2012). Additionally, DNMT3B knockdown depletes H3K27me3 from NC-specific genes to promote their expression and accelerate NCC differentiation (Martins-Taylor et al., 2012). Future studies will need to elucidate the crosstalk between DNA methylation and histone bivalency during lineage commitment and their impact on cell transformation.

DNA methylation also plays a vital role in NB growth and therapeutic response (Fetahu and Taschner-Mandl, 2021). Hypermethylation of CpG islands near promoters–a phenomenon known as the CpG island methylator phenotype (CIMP) – drives epigenetic silencing of tumor suppressors and pro-apoptotic genes across multiple cancer types (Toyota et al., 1999). In NB, CIMP at specific tumor suppressor genes predicts worse patient outcomes (Abe et al., 2005). MNA and non-MNA NBs possess distinct CIMP gene clusters, with MNA-specific CIMP gene clusters enriched for tumor-suppressor genes involved in apoptosis and cell-cycle regulation (Alaminos et al., 2004). Similar to CpG islands, hypermethylation of CpG “shores” has been observed at the regulatory regions of tumor suppressor miRNAs, such as *let-7* miRNAs (Das et al., 2013). Conversely, ablation of CpG methylation in NB cells has demonstrated therapeutic potential, as RA treatment demethylates hypermethylated regions of the *Sox2*-suppressor miRNA-340 and *DNMT1*-suppressor miRNA-152 genes to allow cell differentiation (Das et al., 2013; Das et al., 2010).

### 5.4 RNA modifications

Post-transcriptional RNA modifications (epitranscriptomics), such as RNA methylation, also regulate gene expression by altering mRNA stability, splicing, and localization. Disruption of these modifications depletes NCCs and impairs NCC migration and neural patterning during development (Kim and Jang, 2021). RNA methylation at adenine position N6 (m^6^A), the most prevalent RNA modification, is regulated by several writers, erasers, and readers. Of these, the m^6^A writer methyltransferase (METT) plays a critical role in NC differentiation (Kim and Jang, 2021) and restricts NB oncogenesis through inhibition of *MYCN* expression (Cheng et al., 2020). However, METT also collaborates with MYCN to sustain *HOX* gene expression and an undifferentiated state (Thombare et al., 2024), thus playing opposing roles in NB oncogenesis that must be balanced to maintain a proliferative state. Notably, METLL3 loss ablates NCC formation at the neural plate with failed NC and neuronal cell specification (Kim and Jang, 2021). However, the role of m^6^A readers, writers, and erasers in NB pathogenesis needs further exploration.

## 6 At the crossroads of epigenetics, metabolism, and the TME

### 6.1 Epigenetics and metabolic availability

**FIGURE 3:**
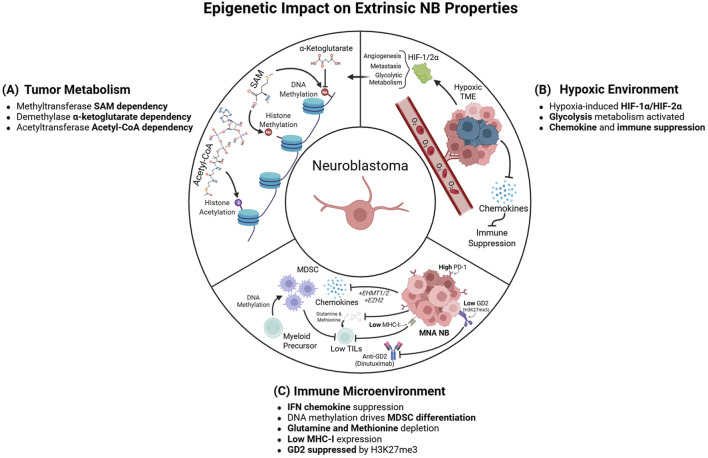
Epigenetic Impact on Extrinsic NB Properties **(A)**. Epigenetic modifications are dependent on the availability of specific metabolites. SAM and acetyl-CoA are critical dependencies for HMTs/DNMTs and HATs, respectively, and DNA demethylases require α-ketoglutarate **(B)**. Hypoxia induces diverse phenotypic changes mediated by epigenetic modifications. Suppression of JMJC-family demethylases by hypoxia drives accumulation of repressive histone marks that block chemokine expression, enforcing immune suppression within the TME. HIF-1α promotes angiogenesis, enables metastasis, and enhances glycolytic metabolism, in turn affecting metabolite-dependent epigenetic changes **(C)**. The NB TME is perturbed to support NB survival, by both NB cells themselves and changes in infiltrating immune cells, including resident MDSCs. T-cell inhibition is enforced by MDSCs and the downregulation of MHC-I on NB cells, and by low chemokine expression due to H3K9me3-and H3K27me3-mediated repression. DNA methylation promotes MDSC formation by repressing transcription of key myeloid genes. Finally, increased H3K27me3 at the GD2 biosynthesis *ST8SIA1* gene obstructs GD2 presentation by NB cells, circumventing anti-GD2 therapies. Created in BioRender https://BioRender.com/InIjvo7.

Crosstalk between epigenetics and metabolism plays a central role during oncogenesis (Supplementary Table S2). Epigenetic changes facilitate metabolic reprogramming during tumorigenesis by promoting the activation or repression of enzymes involved in programs such as fatty acid (FA) and glycolytic metabolism (Ding et al., 2019; Bishayee et al., 2022). In turn, these enzymes modulate the availability of metabolites utilized by epigenetic writers, which can enhance or reduce the deposition of epigenetic modifications, thus altering gene expression (Dai et al., 2020; Huo et al., 2021) (Figure 3A). Histone and DNA methylation rely on methyl groups supplied by one-carbon metabolism, which consists of methionine (Met) and folate cycle. Met, an essential amino acid (AA) that is primarily obtained through diet, is converted into SAM, the methyl-donor substrate for HMTs and DNMTs (Bannister and Kouzarides, 2011). Depletion of Met reduces global histone and DNA methylation in cancer cells and macrophages (Mentch et al., 2015; Dai et al., 2018; Ji et al., 2019) and inhibits NB cell survival (Hu and Cheung, 2009). Combined depletion of Met and folate also downregulates DNMT3A/B expression and blocks proliferation (Poomipark et al., 2016), suggesting that targeting Met metabolism has the potential to block NB tumor growth. On the other hand, indirectly activating Met metabolism via serine supplementation rescues the autophagy induced by H3K9 HMT G9A inhibition (Ding et al., 2013), further demonstrating the dependency of epigenetic regulators on metabolite levels. The impact of Met levels is not limited to the tumor cells themselves: depletion of Met from the tumor microenvironment (TME) via Met transporter upregulation on cancer cells deprives nearby T-cells of Met, inducing histone hypomethylation at the *STAT5* locus and cell death (Bian et al., 2020), indicating that intrinsic Met metabolism also influences the TME.

Metabolic processes also influence the activity of enzymes that remove histone and DNA methylation, notably the TET family of DNA demethylases (TETs) (Kohli and Zhang, 2013) and histone demethylases, including JmjC-domain-containing demethylases (JHDMs) and amine oxidases (LSDs) (Shi, 2007). TETs and JHDMs are dependent on α-ketoglutarate (α-KG) as their substrate; consequently, metabolically-derived analogues of α-KG–including succinate, fumarate and 2-hydroxyglutarate–sterically inhibit their activity (Young et al., 2015; Xiao et al., 2012; Xu et al., 2011). These α-KG-dependent demethylases are critical for maintaining bivalent histone- and DNA-methylation patterns at loci that promote lineage-specific differentiation (Tran et al., 2019). Synthesis and uptake of glutamine (Gln) – an α-KG precursor–is essential for the survival of MYCN-dependent NB (Qing et al., 2012). However, upregulation of α-KG drives NB cell differentiation via DNA methylation depletion (Jiang et al., 2023), suggesting that Gln and α-KG levels must be balanced to maintain growth and avoid DNA hypomethylation-induced growth arrest. Gln and α-KG also control histone methylation, as depletion of Gln and α-KG inhibits JHDMs and blocks H3K27 demethylation, causing hypermethylation of differentiation loci (Pan et al., 2016). Similarly, increasing flavin adenine dinucleotide (FAD) metabolite flux activates LSD1 and promotes differentiation of human neural stem cells (Hirano and Namihira, 2017). However, the impact of LSD dysfunction by FAD flux on NB differentiation has yet to be elucidated.

Acetylation of histones by acetyltransferases also relies on metabolite availability, specifically the transfer of an acetyl group from acetyl-CoA to the ε-amino group of histone lysine (Bannister and Kouzarides, 2011). Acetyl-CoA is primarily derived from glucose metabolism, FA oxidation, and AA catabolism (Shi and Tu, 2015). MYCN exerts global metabolic reprogramming, including activation of pathways contributing to acetyl-CoA synthesis in NB (Oliynyk et al., 2019). Both FA oxidation (Oliynyk et al., 2019) and uptake (Tao et al., 2022) are essential for MNA NB survival. However, the consequences of inhibiting these metabolic dependencies on histone acetylation needs further exploring. Our group and others have also shown that MYCN upregulates the ornithine-to-putrescine enzyme *ODC1* to drive synthesis of polyamines (Hogarty et al., 2008; Tao et al., 2021), a class of arginine- and Met-derived cationic biomolecules that regulate DNA, RNA, and protein stability. Catabolism of the polyamine spermine by SSAT/SAT1 depletes acetyl-CoA (Kee et al., 2004), but the effect of polyamine metabolism on acetyl-CoA and acetyltransferases in NB has not been elucidated. Acetyl-CoA is also depleted under hypoxic conditions due to reduced synthesis from pyruvate (Li et al., 2020). This in turn depletes global histone acetylation and blocks cell differentiation in response to RA, suggesting that acetyl-CoA depletion may provide a benefit to differentiation-resistant tumors. These studies demonstrate that both depletion and enrichment of acetyl-CoA can endow tumors with therapeutic resistance in specific contexts. Similarly, removal of histone acetyl groups is controlled by metabolites. Class I HDACs are inhibited by butyrate and β-hydroxybutyrate (βOHB), which are produced during FA oxidation or ketogenesis (Shimazu et al., 2013). Moreover, βOHB supplementation induces NB apoptosis (Skinner et al., 2009), differentiation (Prasad and Sinha, 1976), and senescence in part through retention of p53 protein acetylation and stabilization (Condorelli et al., 2008). Other metabolites, such as NAD+, are essential for histone deacetylation via Class III HDACs (SIRT1-7) (Lawson et al., 2010) and inhibiting NAD + -generating enzymes in NB downregulates MYCN and obstructs neurosphere formation (Vallejo et al., 2022). Although these studies illustrate the potential for targeting metabolism to control histone acetylation in NB, further work is required.

### 6.2 Epigenetics, metabolism, and the TME

While changes in the metabolic composition of the TME enable NB epigenetic restructuring, these epigenetic changes can in turn regulate the TME (Supplementary Table S3). One known cross talk occurs via hypoxia, a common TME stressor that reorganizes the epigenetic landscape, promoting immune suppression, metabolic reprogramming, and inhibition of cell differentiation (Figure 3B). In NB, hypoxia-inducible factors HIF-1α and HIF-2α are known drivers of growth, angiogenesis, and metastasis (Pahlman and Mohlin, 2018). HIF function is dependent on epigenetic writers: acetylation by HAT1 and p300/CBP stabilizes HIF-1 and HIF-2α, inhibiting proteasomal degradation (Geng et al., 2012; Kumar et al., 2023). This interaction allows HIF-1α to recruit p300/CBP and promote invasiveness (Kim et al., 2022). Class I HDACs likewise stabilize HIF-1α by removing the acetyl group from lysine-532, blocking binding with its cognate ubiquitin ligase (Chang et al., 2011).

NB cell states have differential effects on the surrounding TME. ADRN-NBs are more immunosuppressive and have poor MHC-I expression, low tumor infiltrating lymphocytes (TILs), T-cell exhaustion, and increased infiltration by immunosuppressive cells such as myeloid derived suppressor cells (MDSCs) and tumor associated macrophages (TAMs) (Sengupta et al., 2022). MES tumors possess a more productive immune environment with elevated expression of immunogenic proteins such as natural killer (NK) cell ligands and interferons, and increased recruitment and activation of cytotoxic-T and NK cells (Sengupta et al., 2022). However, MES tumors are more proficient at immune escape from anti-GD2 therapies through H3K27me3-mediated suppression of the GD2 synthesis enzyme *ST8SIA1* (Mabe et al., 2022). Interestingly, forced ADRN-to-MES transition upregulates MHC-I and NK cell-activating ligands, causing T- and NK-cell recruitment (Sengupta et al., 2022). Similarly, PRC2 inhibition in ADRN-NBs upregulates NK ligands and drives NK activity to promote an anti-tumor responses (Sengupta et al., 2022). These data demonstrate that epigenetic modulation of NB cell state can be used to reinvigorate the immune microenvironment and potentiate immunotherapy (Figure 3C).

Metabolic pathways play key roles in regulating epigenetic T-cell activation in the TME. Met uptake is required for T-cells to maintain SAM pool and deposit transcriptionally-activating H3K4me3 at genes controlling T-cell development, so that Met restriction inactivates these genes and suppresses T-cell expansion (Roy et al., 2020). Certain cancers highly depend on Met uptake, limiting Met availability to T-cells (Bian et al., 2020). Similarly, Gln transporter SLC7A5 is required for active T-cell maturation and expansion (Sinclair et al., 2013), and depletion of Gln forces T-cells into the immunosuppressive T-regulatory lineage by remodeling repressive DNA methylation at the Treg-specific enhancer of *FOXP3* (Klysz et al., 2015). Uptake and efflux of these AA by proliferating NB cells may therefore influence T-cell differentiation. Other metabolites are critical in controlling T-cell development; in particular, polyamine metabolism–a pathway used by oncogenic CHAF1A to drive NB growth and differentiation resistance–plays a vital role in enforcing differentiation of T-cells into appropriate lineages, potentially by rewiring global histone acetylation (Puleston et al., 2021). However, it remains unknown whether targeting metabolic pathways controlling AA or polyamine production can alleviate T-cell suppression in the NB TME.

NB TILs display an exhausted phenotype characterized by loss of chromatin compaction, promoter CpG hypomethylation and reduced H3K9/H3K27me3, driving increased transcription across diverse immune-regulatory loci such as the checkpoint ligands *PD-1*, *TIM-3*, and *LAG-3* (Sasidharan Nair et al., 2018). Inhibition of HDAC or BET has been shown to upregulate PD-L1, re-sensitizing cancer cells to immune checkpoint blockade (Zhong et al., 2022; Woods et al., 2015). Furthermore, combination therapies of HDAC or BET inhibitors with PD-1 inhibitors enhance the anti-tumor activity of anti-PD-1 immunotherapy in various cancers including NB (Sauvage et al., 2022; Woods et al., 2015). These studies demonstrate that reversing T-cell exhaustion through epigenetic upregulation of T-cell ligands could reactivate the “cold” TME of NB. Antigen repression is in part induced by MYCN-driven activation of chemokine-repressive genes, such as EHMT1 and EHMT2, which encode GLP and G9a, respectively (Seier et al., 2021). GLP and G9a form a complex that dimethylates H3K9, repressing *IFN-γ* transcription and thereby blocking T-cell responses (Tachibana et al., 2005; Seier et al., 2021). Additionally, MYCN drives EZH2 transcription, which cooperates with GLP/G9a to enforce H3K27me3-and H3K9me2-mediated repression of IFN-γ response (Seier et al., 2021). Consequently, co-inhibition of both EZH2 and G9a induces robust activation of IFN-γ-responsive chemokines, promoting a pro-inflammatory TME (Seier et al., 2021).

Additional NB cell-surface ligands are also subject to epigenetic regulation. The expression of HLA antigens is dependent on histone acetylation at long-range promoters of HLA (Beresford and Boss, 2001), thus it can be upregulated by iHDACs. Similarly, since DNA hypermethylation represses MHC-I expression in cancer cells, treatment with iDNMTs can lead to MHC-I upregulation (Luo et al., 2018; Cornel et al., 2020). In particular, entinostat has been shown to upregulate MHC-I, NK-related receptor ligands, and immunoproteasome subunits, overall promoting active T-cell effector functions in NB (Cornel et al., 2022). Therefore, targeting epigenetic factors is an attractive therapeutic option to promote clearance of NB cells by enhancing T- and NK-cell immune responses. Dinutuximab has revolutionized NB treatment, increasing patient therapeutic response (Voeller and Sondel, 2019). However, GD2 expression is heterogeneous, and many patients will relapse. Dinutuximab resistance can be reversed via PRC2 inhibition, which decreases H3K27me3 and increases H3K4me3 at the promoter of *ST8SIA1* – which converts GM3 to GD3 in GD2 biosynthesis–thus promoting GD2 expression and re-sensitizing NB to anti-GD2 therapy (Mabe et al., 2022). This sensitization is in part driven by a MES-to-ADRN transition subsequent to EZH2 inhibition, which reduces repressive H3K27me3 and upregulates ADRN-specific genes (Mabe et al., 2022). Therefore, in addition to epigenetic inhibitors, exploiting ADRN/MES cell states has the potential to improve antigen presentation and treatment response to anti-GD2 and other immune-based therapies.

## 7 Discussion

While the mechanisms driving NB oncogenesis from NC derivatives remain incompletely characterized, our understanding of the events preceding NC transformation have provided insight into specific developmental stages whose dysregulation contributes to disease onset. Among the primary oncogenic drivers, MYCN has been shown to control both NC differentiation and migration as well as drive *de novo* NB formation, while additional chromatin remodelers such as the SWI/SNF complex and CHAF1A have demonstrated emerging roles in blocking NC differentiation and driving NB tumorigenesis, in part through metabolic rewiring of the polyamine synthesis pathway. However, more work is needed to determine whether targeting chromatin remodelers can improve RA sensitivity, tumor differentiation, and clinical patient outcomes.

Epigenetics play a vital role in NC development: distinct patterns of histone acetylation and methylation, as well as DNA methylation, define the active enhancers and genes in a given cell type, which in turn restrict NCCs to distinct lineage fates. By elucidating the transcriptional and epigenetic changes in developing NCCs, single-cell and lineage tracing studies have provided novel insights into the NC lineage restriction process and have permitted the identification of intermediate cell states during NC development. Such studies have uncovered the most probable cells of origin for NB, namely, neuroblasts and SCPs, which participate in a coordinated dynamic between the descendants of distinct NCC populations in the trunk to form nerve and chromaffin components of the SA system. Future studies may improve personalized therapy by stratifying NB patients according to their expression of key dysregulated epigenetic programs in relation to their degree of differentiation, cell-state plasticity, and MYCN amplification, thus enabling precise targeting of the epigenetic transcriptome remodeling underlying each tumor’s initial transformation and ongoing disease progression.

NB cells express many of the same SA regulatory factors as their NC progenitors, and the expression and epigenetic enhancer states at specific SA-related genes collectively define CRCs unique to ADRN and MES NB states. These states enforce disparate tumor properties, with ADRN favoring a fast-growing but chemo-sensitive behavior, while MES exhibit more indolent growth and therapy resistance combined with elevated immune infiltration. While the phenotypic differences between these states are well understood, their distinct susceptibilities to chemotherapy, differentiation, and immune attack have yet to be exploited in combination with targeting the epigenetic factors that control NB’s ability to regulate these properties, which may block them from ultimately adapting and developing therapy resistance. Obstructing the CRC switches that permit ADRN-to-MES and reverse transitions could also curtail any resistance derived from dynamic cell-state heterogeneity in these tumors.

Epigenetic changes in NB can also drive metabolic changes to support NB growth, in conjunction with MYCN, including the rerouting of FA and glycolytic pathways. These epigenetic modifications are themselves dependent on metabolic processes such as SAM, α-KG and acetyl-CoA metabolism to provide their substrates. Epigenetic repression additionally obstructs immunogenicity of the NB TME, exemplified by MYCN-driven activation of H3K9 methyltransferases that silence the interferon pathway for T-cell recruitment. Future clinical paradigms for NB may include treatments designed to forcibly shift ADRN/MES phenotypes to re-invigorate the immune microenvironment, or to re-sensitize these tumors to chemotherapy and differentiation.

Because epigenetic changes have been implicated in regulating such diverse steps in NB oncogenesis and progression, inhibiting the epigenetic proteins responsible for these changes has become a priority for targeted therapy in NB. Pre-clinical studies have shown that histone modification writers, erasers, and readers alike can be exploited to kill NB cells. However, NB epigenetic clinical trials remain limited. Future translational trials will likely advance our understanding of how epigenetic therapies can suppress NB tumor growth, both independently and as augmentation of existing treatments to improve NB patient outcomes.
