# Supplementary material for: Epigenetic crosstalk in neuroblastoma development and progression
Source: Front Cell Dev Biol. 2026 Mar 11;14:1769372. doi: 10.3389/fcell.2026.1769372 (PMC13013432; doi:10.3389/fcell.2026.1769372)
Supplement: Supplementary file 1 [file Table1.docx]

**Supplementary Tables**

**Supplementary Table 1. Core developmental pathways perturbed in NB oncogenesis.**

| **Process** | **Development Function** | **Oncogenic Function** | **Key Regulators** | **References** |
| --- | --- | --- | --- | --- |
| Cell Cycle Progression | Promotes expansion of intermediate progenitors prior to differentiation | Accelerates tumor growth and mitigates “brakes” limiting cell-cycle progression | - *E2F1* - *MKI67* - *TOP2A* - *UBE2C* | - Bonine et al., 2024 - Grossmann et al., 2024 |
| DNA Replication Stress Response | Resolve DNA damage caused by mechanical strain on unwinding DNA during S-phase | Enables faster S-phase progression for cell proliferation | - *ATR* | - Bonine et al., 2024 |
| Cellular Stress Response | Protect the cell from DNA-damaging oxidative species and other reactive toxins | Evade chemotherapy-induced cell death | - *MTOR* - *NFKB* family - *ATF3* - *TNF* | - Grossmann et al., 2024 |
| Migration & Growth | Required for “bridge” transitional state from SCP to chromaffin identity | Drives aberrant cell proliferation and metastatic invasion | - *E2F7* - *TWIST1* | - Xu et al., 2025 |

**Supplementary Table 2. Epigenetic crosstalk with metabolism in NB oncogenesis.**

| **Epigenetic Feature** | **Metabolic Dependencies** | **Points of Crosstalk** | **Impact on NB** | **References** |
| --- | --- | --- | --- | --- |
| DNA Methylation | - SAM synthesis - One-carbon Met cycle - One-carbon folate cycle | - Depletion of Met and folate blocks DNA and histone methylation | - Met depletion blocks NB proliferation, synergizes with chemotherapy-induced cell death | - Mentch et al., 2015 - Dai et al., 2018 - Ji et al., 2019 - Hu and Cheung, 2019 |
| Histone Methylation |  |  |  |  |
| DNA De-Methylation | - α-ketoglutarate (α-KG) - Glut and Citric Acid cycle | - α-KG -like metabolites antagonize DNA and histone demethylase activity | - Depletion of α-KG precursor (Glut) kills MYCN-driven NBs - Excessive α-KG enforces differentiation | - Xiao et al., 2012 - Xu et al., 2011 - Pan et al., 2016 - Qing et al., 2012 - Jiang et al., 2013 |
| Histone De-Methylation |  |  |  |  |
| Histone Acetylation | - Acetyl-CoA - FA oxidation and uptake - AA catabolism | - Low Acetyl-CoA depletes global histone acetylation | - FA depletion blocks cell proliferation and tumor growth - Low Acetyl-CoA blocks RA-induced differentiation | - Li et al., 2020 - Oliynyk et al., 2019 - Tao et al., 2022 |
| Histone De-Acetylation | - NAD+ - FA oxidation and ketogenesis | - NAD+ loss inactivates sirtuin HDACs - FA byproduct (β-hydroxy-butyrate) inhibits HDACs | - NAD+ depletion blocks neuro-sphere growth and induces apoptosis - β-hydroxy-butyrate induces growth arrest and apoptosis | - Lawson et al., 2010 - Shimazu et al., 2012 - Vallejo et al., 2022 - Skinner et al., 2009 - Condorelli et al., 2009 |

**Supplementary Table 3. Epigenetic crosstalk with the TME in NB oncogenesis.**

| **Epigenetic Feature** | **TME Impact** | **Points of Crosstalk** | **Impact on NB** | **References** |
| --- | --- | --- | --- | --- |
| Histone Acetyltransferases (HAT1, p300/CBP) | - Hypoxia response (HIF-1/2α) | - Histone HATs and HDACs control HIF-1/2 protein stability | - HIF-1α and HIF-2α enable NB survival, angiogenesis, and metastasis | - Geng et al., 2012 - Kumar et al., 2023 - Chang et al., 2011 - Påhlman et al., 2018 |
| Histone De-Acetylases (Class III HDACs) |  |  |  |  |
| ADRN-specific Epigenome (H3K27ac at ADRN enhancers) | - Low MHC-I and NK cell ligands - Low TILs - High MDSCs and TAMs | - H3K27me3- mediated repression of immune genes | - Poor MHC-1 presentation prohibits T-cell-induced apoptosis - MDSC/TAM recruitment neutralizes cytotoxic T-cells - ADRN to MES switch enables T- and NK-cell recruitment | - Sengupta et al., 2022 |
| MES-specific Epigenome (H3K27ac at MES enhancers) | - High MHC-I and NK cell ligands - Interferon and inflammatory signaling - High TILs - Low GD2 | - MES CRC gene PRRX1 upregulates MHC-I - H3K27ac/H3K4me3 activates immune genes - H3K27me3-mediated repression of GD2 | - MHC-I, NK ligands, and immune signaling recruit T-cells - Poor GD2 expression is reversed by H3K27 methyltransferase inhibition | - Sengupta et al., 2022 - Mabe et al., 2022 |
| T-cell Epigenome (Histone & DNA modification at T-cell lineage genes) | - Required for T-cell development - Controls lineage-specific differentiation | - Low SAM blocks H3K4me3-mediated activation of T-cell development genes - Low Glut redistributes DNA methylation to favor Treg differentiation - Loss of DNA methylation and H3K9/H3K27me3 at checkpoint loci drives T-cell exhaustion | - SAM tumor uptake restricts pro-differentiation H3K4me3 deposition in T-cells - Glut tumor uptake promotes Treg lineage - Inhibiting H3K9 HMT and chromatin remodelers restore T-cell ligand expression | - Roy et al., 2020 - Bian et al., 2020 - Klysz et al., 2015 - Sasidharan Nair et al., 2018 - Sauvage et al., 2022 - Seier et al., 2021 |
